# Peripheral Intravenous Catheter Complications: A Comparative Analysis of 6 Reference Documents

**DOI:** 10.1097/NAN.0000000000000640

**Published:** 2026-05-08

**Authors:** Baudolino Mussa, Noemí Cortés Rey, Fredericus H. J. van Loon, Gema Munoz Mozas, Vincent Piriou

**Affiliations:** **Author Affiliations:** Surgical Science Department, University of Turin, Torino, Italy (Mussa); A Coruña and Cee Health Area, Hospital Teresa Herrera, Complejo Hospitalario Universitario de A Coruña As, Xubias de Abaixo, A Coruña, Spain (Cortés Rey); Faculty of Perioperative Care and Technology, University of Applied Sciences, Catharine Hospital, Fontys University of Applied Sciences, Eindhoven, The Netherlands (van Loon); Royal Marsden NHS Foundation Trust, Chelsea, London, UK (Munoz Mozas); Hospices Civils de Lyon, Université Claude Bernard Lyon-1, Villeurbanne, France (Piriou).

**Keywords:** guidelines, patient safety, peripheral intravenous catheters, standardized terminology, vascular access device complications

## Abstract

**Objective:**

The objective of this study was to analyze current evidence-based pan-European standards of good clinical practice in the use of peripheral intravenous catheters (PIVCs) to determine if they meet clinicians’ needs for unambiguous, standardized guidance for the prevention and management of PIVC-associated complications.

**Methods:**

Reference documents meeting agreed inclusion criteria were identified and analyzed. Documents were systematically searched for terms (selected based on expertise of the authors and supported by scientific literature) most likely to be associated with the prevention/management of complications associated with the use of PIVCs. A matrix was constructed to visualize the degree of consensus across guidelines regarding prevention and management terminology. Recommendations for optimizing clarity and standardization across Europe were proposed.

**Results:**

Few European reference documents included PIVCs within their scope, and few were subject to a quality review. Adherence to the established GRADE framework of rating supporting evidence was missing from all reference documents. There was ambiguous terminology across documents and many instances of omission of terms rated important by the authors for the prevention and management of complications.

**Conclusion:**

An English-language European guideline for the prevention of complications associated with PIVCs is needed, featuring consensus terminology and a standardized, systematic, and transparent method for grading evidence.

## BACKGROUND

Peripheral intravenous catheters (PIVCs), including short peripheral catheters, long peripheral catheters, and midline catheters, are the most commonly used intravenous (IV) devices in clinical practice and represent one of the most frequent invasive procedures performed in health care settings.^1^

Reported complication rates range from 35% to 50%, although inconsistent terminology and definitions across studies may contribute to this variation. Local complications, such as phlebitis, catheter dislodgement, infiltration, extravasation, and catheter-related bloodstream infections, are common and increase the economic and clinical burden on patients, caregivers, and health care systems.^2^ The frequency of complications also contributes to higher treatment costs and prolonged hospital stays.

Ensuring patient safety requires that clinicians are competent in preventing complications, recognizing symptoms, selecting appropriate interventions, and minimizing PIVC-related harm. To support this, best-practice standards for the prevention, assessment, and management of complications have been consolidated in various reference documents.

The Institute of Medicine defines a clinical practice guideline as “statements that include recommendations, intended to optimize patient care, that are informed by a systematic review of evidence and an assessment of the benefits and harms of alternative care options.”^3^ Such guidelines must include (1) a systematic review of the research evidence addressing a specific clinical question and the strength of that evidence, and (2) a set of recommendations that integrate the evidence with value judgments regarding the benefits and harms of alternative care options.^3^

Currently, no English-language European guideline exists for the prevention of PIVC-related complications that applies a consistent terminology and a systematic, transparent process for assessing evidence.

## METHODS

### Authors

The authors who conducted and authored the study comprised 5 senior vascular access health care professionals (anesthetists, interventional radiologists, lead IV nurses, and surgeons) from 5 European countries. They have been lead authors on numerous peer-reviewed publications in the vascular access literature and have spoken at vascular access congresses nationally and internationally. These authors have experience in founding national vascular access societies, training vascular access nurses at the certified diploma level, and contributing to guidance preparation.

In 2021, the authors began exploring the need for a consensus document, based on various European reference documents, for the prevention and management of PIVC-related complications. Between December 2021 and December 2024, the authors convened several virtual and face-to-face meetings to systematically analyze these reference documents.

### Selection of Reference Documents

The authors defined inclusion criteria to identify reference documents focused on PIVCs. No publication timeframe was applied, as guidance specific to PIVCs has only recently become a focus of attention. Only documents available in English were included because evidence-grading frameworks, such as the GRADE (Grading of Recommendations, Assessment, Development, and Evaluation) Working Group, typically rely on English-language sources.^4^ Accordingly, the analysis was conducted using English-language search terms.

Additional inclusion criteria included the following: documents must represent nationally adopted guidance; scope must include PIVCs; guidance must originate from a European Union (EU) country; documents must have undergone a defined quality review process; and guidance must be evidence-based rather than purely consensus-based.

A comprehensive search identified 12 European reference documents (Table 1). Of these, 5 met all inclusion criteria and were selected for comparative analysis: the European Recommendations for Proper Indication and Use of Peripheral Vascular Access Devices (ERPIUP); Commission for Hospital Hygiene and Infection Prevention (KRINKO); Clinical Practice Guideline on Intravenous Therapy with Temporary Devices in Adults (CPG); National Evidence-Based Guidelines for Preventing Healthcare-Associated Infections in Hospitals (epic3); and Standards for Infusion Therapy (RCN).^1,5-9^

**TABLE 1:** Guideline/Reference Documents Considered for Analysis

Origin	Title	Year of Publication	Available in English	Nationally or Internationally Adopted	Peripheral VADS Included in Scope	European Focus	Result of Defined, Quality Review Process	Evidence Based or Consensus	Selected for Study Comparative Analysis
Europe	ERPIUP (European Recommendations for Proper Indication and Use of Peripheral Vascular Access Devices)	2021	Yes	Yes	Yes	Yes	Yes	Evidence based and consensus	Yes
France	SF2H (French Society for Hospital Hygiene)	2019	No	Yes	Yes	Yes	Yes	Evidence based	No
Germany	KRINKO (Commission for Hospital Hygiene and Infection Prevention)	2017	Yes	Yes	Yes	Yes	Yes	Evidence based	Yes
Italy	Minerva Medica	2018	Yes	No	Yes	Yes	Yes	Consensus	No
Italy	SIAARTI (Italian Society of Anesthesia, Analgesia, Resuscitation and Intensive Care)	2020	No	No	Yes	Yes	No	Consensus	No
Spain	CPG (Clinical Practice Guideline)	2014	Yes	Yes	Yes	Yes	Yes	Evidence based	Yes
Spain	Phlebitis Zero	2018	No	No	Yes	Yes	No	Consensus	No
Spain	SEIMC (Spanish Society of Infectious Diseases and Clinical Microbiology)	2016	No	No	Yes	Yes	No	Consensus	No
UK	epic3	2014	Yes	No	Yes	Yes	Yes	Evidence based	Yes
UK	NIVAS (National Infusion and Vascular Access Society)	2021	Yes	No	No	Yes	No	Consensus	No
UK	RCN (Royal College of Nursing)	2016	Yes	Yes	Yes	Yes	Yes	Evidence based	Yes
USA	INS^a^ (Infusion Nurses Society)	2024	Yes	Yes	Yes	No^a^	Yes	Evidence based and expert consensus	Yes

Abbreviations: CPG, Clinical Practice Guideline; ERPIUP, European Recommendations for Proper Indication and Use of Peripheral Vascular Access Devices; INS, Infusion Nurses Society; KRINKO, Commission for Hospital Hygiene and Infection Prevention; NIVAS, National Infusion and Vascular Access Society; RCN, Royal College of Nursing; SEIMC, Spanish Society of Infectious Diseases and Clinical Microbiology; SF2H, French Society for Hospital Hygiene; SIAARTI, Italian Society of Anesthesia, Analgesia, Resuscitation and Intensive Care.

a Although INS is not European focused, it is adopted as a standard by many European countries that lack their own guidance. Therefore, for the purpose of this study, INS was chosen by the Reviewers to be included as a reference document.

Although one of the inclusion criteria specified that guidance should originate from an EU country, the Infusion Nurses Society’s (INS) *Infusion Therapy Standards of Practice* were also included. Despite being developed in the United States, the INS *Standards* are widely adopted or referenced in many European countries that lack their own national guidance. Their inclusion was, therefore, considered essential to provide a comprehensive and comparable overview of evidence-based standards relevant to PIVC management in Europe.

This selection process ensured that the analysis focused on the most relevant and widely implemented guidelines for the prevention and management of PIVC-related complications across Europe.

### Complications

The authors selected 9 PIVC complications to examine on the basis of common occurrence: occlusion, catheter-associated bloodstream infection (CABSI), local infection, dislodgement, phlebitis, thrombophlebitis, thrombosis, extravasation, and infiltration.^1^

### Terms Associated With Complication Characteristics

The authors identified 46 terms that, with their expertise and, as supported by the scientific literature, are most likely to be associated with the prevention and management of the target PIVC complications (Table 2).

**TABLE 2:** Terms Associated With Complication Characteristics

Aseptic technique(s)	PIVC extension
Catheter length	PIVC size
Catheter material	Pre-filled syringes
Catheter stabilization	Record keeping
Catheter/vein ratio	Reinforced dressing
Checklists/bundles	Removal
Closed systems	Scrub the hub
Connectors	Securement device
Dressing	Site location
Drug compatibility	Site selection
Extension sets	Skin antisepsis
Extension to an infusion system	Sass
Flushing	Sutureless securement device
Glue	Sutures securement device
Hair removal	Therapy
Infusion pressure	Thrombolytics
Infusion set replacement	Training/education
Kinking	Transparent dressing
Locking	Ultrasound
Monitoring	Vascular access team
Multiple placements	Vein selection
Multiple punctures	Vesicant/irritant/osmolarity/ph
Pistoning/pumping (avoid)	When to remove

Abbreviations: PIVC, peripheral intravenous catheter; SASS, subcutaneous anchorage securement system.

### Process

Each of the reference documents was searched for a connection between the 9 complications and the 46 terms (Table 2). Relevant text was extracted from each reference document and inserted into an analysis matrix. This search was conducted completely independently of the authors’ assignment to avoid bias and provide a comprehensive view of what the reference documents included.

To determine importance, a score of “1” was allocated to terms linked to prevention/management of a complication by authors, with an importance score of “0” instead allocated for terms not deemed to be associated with complication prevention/management by authors. Terms linked to prevention/management of a complication by each reference document were qualified as “included” and received an inclusion score of “1/6” per document. The term-to-complication connection was subsequently determined through the addition of importance and inclusion scores. For example, checklists/bundles were linked to the prevention of local infection by the authors, resulting in an importance score of 1, and were also linked in 4 of the documents, generating an inclusion score of 4/6. Adding these scores yielded a term-to-complication connection of 1.7. Similarly, checklists/bundles were linked to the prevention of phlebitis by the authors and were also linked in 1 of the documents. The resulting importance score of 1 and inclusion score of 1/6 combined to generate a term-to-complication connection of 1.2.

Term-to-complication connections fell into one of 4 categories. The “important and included” category indicated the term-to-complication to be important and included in at least 1 selected reference document (ie, what the documents *do* include is corroborated by the authors). “Important but not included” specified connections to be important but not included in at least 1 selected reference document (ie, what the documents *do not* include, but perhaps should, based on authors’ consensus). Term-to-complication connections which were deemed not important but still included in at least 1 selected reference document were allocated to the “not important but included” subgroup (ie, what the documents do include, but the authors did not consider to be relevant in practice), while those which were neither important nor included in at least 1 selected reference document were grouped as “not important nor included” (ie, terms that were not connected/considered important to the complication by either the documents or the authors).

## RESULTS

### Analysis of Term-to-Complication Connections

There were 138 term-to-complication connections that were determined to be important and included (Table 3).

**TABLE 3:** Analysis of Term-to-Complication Connections

Reference Document Mentions	Reviewer Indication		
		Important	Not important
	At least 1	138	54
	None	88	134

**Figure 1.**
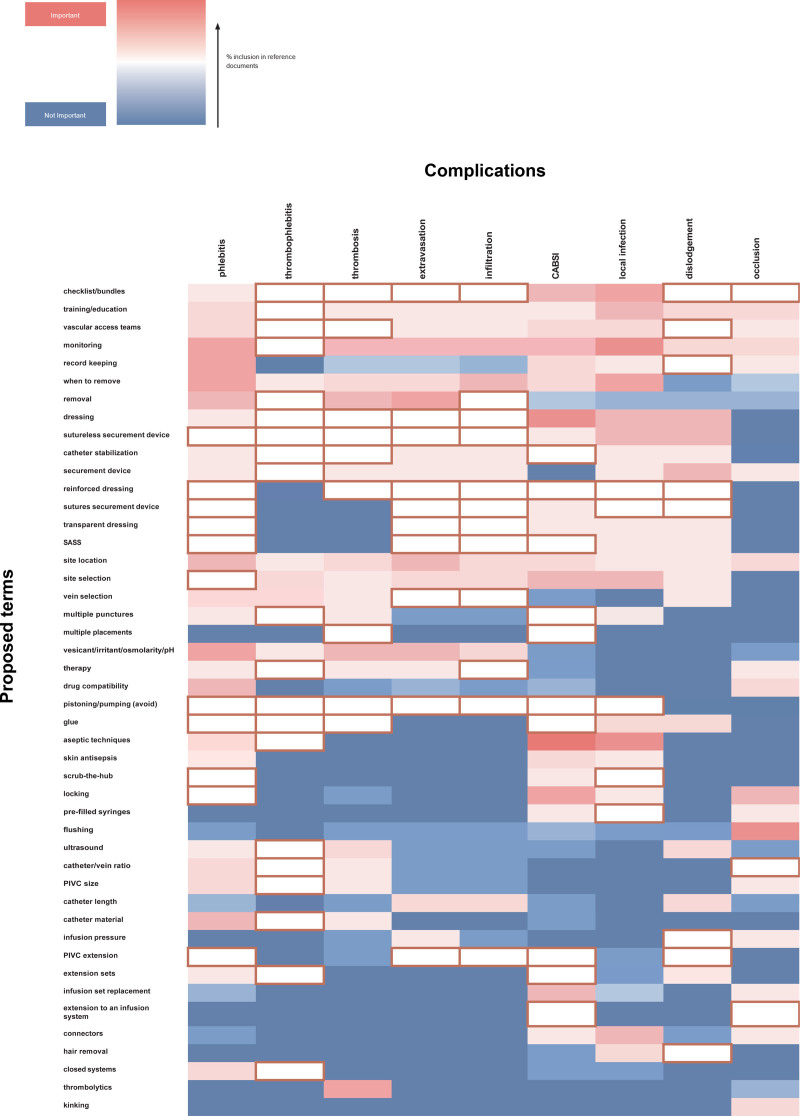
Heat map of term-to-complication connection analysis. Cells outlined in dark red denote term-to-complication connections that were determined to be important but not included in any reference document—enabling at-a-glance clarity of areas of gaps or limited coverage across documents. *Abbreviations: CABSI, catheter-associated bloodstream infection; PIVC, peripheral intravenous catheter; SASS, subcutaneous anchorage securement system.*

There were 88 term-to-complication connections that were determined to be important but not included. These cells are outlined in dark red in the heat map, to denote their at-a-glance significance to this analysis, aiding quick and easy identification of areas of gap or limited coverage across reviewed documents (Figure 1). By presenting the information in this format, readers can easily identify which core topics are well addressed and which remain insufficiently developed. This visual summary is intended to facilitate future efforts to produce a more complete and harmonized document that covers the most essential aspects of vascular access practice. Note: this does *not* mean that the reference documents do *not* mention the term at all; rather, there is no clear connection drawn between the term and the prevention/management of one of the focused complications.

There were 54 term-to-complication connections that were determined to be not important but that were included and a further 134 term-to-complication connections that were determined to be not important nor included.

Term-to-complication connections showed notable trends across complications and terms. Using the important/inclusion score for each potential term-to-complication connection, a heat map and clustered rows/columns by average score was generated (Figure).

Red cells were noted as linked (important) by the authors and blue were not linked (not important): dark red indicates important and included in many reference documents; light red indicates important but not included in many or any reference documents; light blue indicates not important and included in some of the reference documents, and dark blue indicates not important and not included. Dark cells on the heat map indicate strong consensus, while light cells indicate weak consensus.

The indicative parts of the heat map are the dark red to light red and light blue areas.

In terms of assessing whether term-to-complication connections were strong or moderate or weak, the following breakdown was applied:

- Strong: Connections in 3 or more reference documents (a score of 1.5-2 on the heat map when term was important and 0.5-0.8 if not important).
- Moderate: Connections in 2 reference documents (a score of 1.3 if important and 0.3 if not important).
- Weak: Connections in 1 or no reference documents (a score of 1-1.2 if important and 0-0.2 if not important).

Analyzed in terms of strong, moderate, and weak connectivity, the term-to-complication connections are grouped in Table 4.

**TABLE 4:** Connectivity per Complication

Complication	Strong	Moderate	Weak
Phlebitis	**8**	8	30
Thrombophlebitis	-	2	**44**
Thrombosis	4	4	**38**
Extravasation	5	4	37
Infiltration	2	5	**39**
CABSI	**8**	7	31
Local infection	**10**	4	32
Dislodgement	3	6	37
Occlusion	3	6	37

Abbreviation: CABSI, catheter-associated bloodstream infection.

When similar word searches were grouped, it was clear that, while broadly associated with the same complications, there were variations. Examples include “when to remove” and “removal,” “site location” and “site selection,” and “aseptic techniques” and “skin antisepsis.” These observations support the importance of the standardization of the search terms and the language around the complications with which the reference documents might better be interrogated.

Connections between the prevention and management of complications and any one of the elements of the vesicant/irritant/osmolarity/pH compound search term were recorded as a positive hit. As implicated in its listing adjacent to “therapy” and “drug compatibility” search terms, it was considered by the authors to be used in the context of a general search on the properties of the infusate.

## DISCUSSION

### Comparative Analysis of Reference Documents

A key consideration in this study’s search methodology was that different reference documents used alternative terminology, which could limit the effectiveness of key word searches. This variability often resulted from differences in translation into English. To address this, the authors refined the search strategy by including common term variations when expected content was not initially found. Including these variations did not change the overall findings or conclusions about consensus.

Nevertheless, this finding highlights the problem of inconsistent terminology rather than diminishing its importance. The coexistence of terms such as catheter-related thrombosis and catheter occlusion to describe similar complications illustrates the lack of linguistic standardization across reference documents. Such inconsistencies hinder communication among health care professionals, delay clinical decision making, and compromise patient safety. They also make training, outcome comparison, and research synthesis, such as meta-analyses, more difficult.

The results emphasize the urgent need to standardize terminology related to PIVC complications. Future guidelines should not only provide evidence-based recommendations but also establish a universally accepted nomenclature to enhance clarity, comparability, and patient outcomes.

It was challenging to identify European reference documents that fully met the inclusion criteria. Some documents remain in use despite not having been updated for more than a decade. The lack of a common European language also contributes to the limited availability of guidance and complicates key word searches in translated documents.

Of the 12 documents identified, 5 met all inclusion criteria: ERPIUP, KRINK, CPG, epic3, and RCN. Although one criterion specified that documents should originate from a European Union country, the INS *Infusion Therapy Standards of Practice* (*Standards*) were also included. Despite being developed in the United States, the INS *Standards* are widely adopted or referenced in European countries lacking national guidance. Their inclusion provided a meaningful benchmark for comparison, given their completeness and international influence.^9^

Among the 6 reference documents analyzed, the INS *Standards* contained the highest proportion of expected content, followed by the ERPIUP/World Congress on Vascular Access (WoCoVA) Consensus.^1,9^ The recency of these documents (published in 2024 and 2021, respectively) may explain their closer alignment with the most up-to-date evidence and with the authors’ expectations. Among the national documents, the UK Royal College of Nursing Standards for Infusion Therapy demonstrated the strongest consistency with expected content.^8^ The remaining documents showed variable alignment, with fewer references to specific complication-related terminology.

Across all documents, there was noticeable variation in the number of terms used for complication prevention and management. Out of 46 terms analyzed, the distribution was as follows: 30 for CABSI, 28 for phlebitis, 27 for local infection, 23 for thrombosis, 21 for occlusion, 19 for dislodgement, 20 for extravasation, 18 for infiltration, and 5 for thrombophlebitis. Each document showed relative strengths in specific areas; for example, ERPIUP in dislodgement, local infection, and phlebitis; CPG in local infection; epic3 and RCN in CABSI and extravasation; and INS across most categories. This variation reflects the differing scope and focus of each reference document.

None of the 6 documents were developed using the systematic and transparent framework of GRADE for rating the quality of evidence. GRADE provides a structured approach for developing clinical practice recommendations, categorizing evidence certainty from high to very low. When the certainty of evidence is low, recommendations are likely to be weak, and vice versa. A more systematic approach, based on GRADE or similar reputable platforms, would strengthen future guideline development and ensure clearer, more reliable recommendations.^4,10,11^ It should be noted, however, that the widely used INS *Standards* employed a recognized, reputable, alternate health care platform for clinical evidence appraisal, with use of the Ovid Synthesis Clinical Evidence Manager and the Strength of the Body of Evidence rating scale.

### Analysis of Term-to-Complication Connections

The standout finding from this analysis was that, of the 226 term-to-complication connections agreed by the authors to be important, 88 were not found in any of the reference documents. At least one of these cells was found in every complication, including those for which there was the greatest concordance between importance and inclusion. The search terms with the highest consensus between authors’ expectations and reference documents were monitoring, record keeping, site selection, and when to remove. The highest number of reference documents citing term-to-complication connections was found for aseptic techniques and dressings (CABSI), aseptic techniques and monitoring (local infection), and flushing (occlusion).

The strongest connectivity between author expectation and reference documents citation was found for local infection, phlebitis, and CABSI (>45 connections); the weakest were infiltration, thrombosis, and thrombophlebitis (possibly owing to perceived ambiguity in the reference documents as to the identification of thrombophlebitis as a distinct PIVC complication). Weak consensus predominated all complications.

### Alternative Terminology

A feature of the search methodology was the possibility of alternative terminology being used in reference to a complication, which would mean a key word search would not find that relevant content. The possibility of alternative terminology may also be the consequence of variability in the translation of reference documents into English. A review of the initial search results was necessary to account for this and strengthen any determination on consensus. Once the key word search data had been accrued and analyzed, it was reassessed by the authors to identify refinements to the search terms. Where the authors were expecting to find content in relation to the complication but did not, common variations were generated and put through another key word search. In no instance was there seen to be an impact of including variable terminology in the search material to the overall findings.

Reference documents that included the highest proportion of expected content were the INS *Standards*, followed closely by the ERPIUP/WoCoVA Consensus.^1,9^ Nevertheless, none of these are guidelines, strictly speaking.^3^

The study identified several instances of variable consensus between the reference documents chosen for this study and in terms of consensus with authors’ expectations.

While variable terminology was identified and factored into analysis (and this does raise queries around the impact of variations on standardization in care), these variations did not noticeably impact the overall level of consensus observed. There was evidence in the study, however, of confusion in terminology in the reference documents (eg, catheter-related thrombosis and catheter occlusion).

Inclusion in some reference documents of terms deemed not important in relation to particular complications suggests that they may not contain the most up-to-date evidence. Similarly, the study also identified many instances where the documents did not explicitly make a connection between a term and the prevention/management of a complication, as had been expected by the authors.

The focus on determining consensus across a wide selection of literature created the need to limit the accrual of data to a key word search of the selected reference documents instead of extensive line-by-line analysis. This limits the assessment of the content to a binary issue of whether or not there was content in relation to a complication and does not address the details of the content. This, however, does not exclude the objective of determining a broad level of consensus across the selected reference documents, which allows for further detailed analysis.

### How to Proceed

The authors’ call to standardize terminology is both feasible and necessary, given the global use of PIVCs. Such standardization would align with existing global frameworks like CPR algorithms and the increasingly adopted ANTT® technique.^12,13^ This goal could be achieved through a structured Delphi-style survey involving vascular access experts from various countries, inviting broad participation in this valuable initiative. Engagement from multiple professional societies would foster support and lead to a Consensus Conference to finalize the terminology.

The article’s contribution to resolving inconsistencies in PIVC guidelines is significant for several key reasons.

### Patient Safety Impact

Inconsistent terminology can lead to miscommunication between health care providers. Misunderstandings about complications and their management could result in delayed or incorrect interventions. Standardized terms are crucial for accurate reporting and monitoring complications.

### Evidence-Based Practice Challenges

The study found 88 important term-to-complication connections that were not included in any reference documents. This gap means that critical preventive measures or management strategies might be overlooked, as health care providers may miss important connections between practices and complications.

### Clinical Training and Education

Variable terminology makes it difficult to train new health care professionals consistently, develop standardized protocols, compare outcomes across different facilities, and implement quality improvement initiatives.

### Documentation and Research Impact

The study revealed confusion in terminology. This makes it difficult to conduct accurate meta-analyses, compare studies across different institutions, aggregate data for large-scale research, and establish clear best practices.

## LIMITATIONS

There are 4 principal limitations to this study. First, in the absence of a validated terminology by which to refer to complications and their associated terms, the authors, although experts in vascular access management, were necessarily subjective in their analysis of the reference documents. Second, the methodological heterogeneity of the reference documents made it impossible to compare their recommendations regarding prevention and management of complications. Third, divergence in non-standardized terminology is exacerbated through translation of national reference documents into English. Fourth, 4 of the 6 reference documents in use have not been updated in more than 8 years: even if terminology were standardized, these documents would not convey up-to-date vascular access management.

## CONCLUSION

The analysis revealed that few European reference documents include PIVCs, existing documents lack systematic quality review, none fully meets clinical practice guideline standards, and there is variable consensus in terminology across documents. Standardization would have many practical implications, including improved communication between health care providers, enhanced patient quality care, facilitation of better research and data collection, enabling of more effective training programs, and support of evidence-based practice implementation.

The article emphasizes the need for consensus-building among experts, clear definition of terms, explicit connection between terms and complications, regular updates based on new evidence, and translation into multiple languages, while maintaining consistency.

A single English-language European guideline (from which translations can be made available) for the prevention of complications associated with PIVCs, featuring consensus terminology and a standardized, systematic and transparent method for grading evidence, is definitively warranted and achievable.

Such a guideline would align with recently published calls to action for a single, global, most recent evidence-based standardization of best practice in the management of PIVCs, and for universally accepted terminology in vascular access management worldwide.^14,15^

This article’s findings underscore how critical it is to establish standardized terminology in future guidelines to ensure better patient outcomes and more effective health care delivery.

## References

[1] Pittiruti M Van Boxtel T Scoppettuolo G. European recommendations on the proper indication and use of peripheral venous access devices (the ERPIUP consensus): a WoCoVA project. J Vasc Access. 2023;24(1):165-182. doi:10.1177/11297298211023274 34088239 10.1177/11297298211023274

[2] Helm RE. Accepted but unacceptable: peripheral IV catheter failure: 2019 follow-up. J Infus Nurs. 2019;42(3):149-150. doi:10.1097/NAN.0000000000000324 30985564 10.1097/NAN.0000000000000324

[3] Institute of Medicine (US). Committee on standards for developing trustworthy clinical practice guidelines. In: Graham R Mancher M Miller Wolman D, eds. Clinical Practice Guidelines We Can Trust. Washington, DC, USA: National Academies Press; 2011. doi:10.17226/13058 24983061

[4] Siemieniuk R Guyatt G. What is GRADE? BMJ best practice. Published 2025. Accessed August 27, 2025. https://dev-bestpractice.bmjgroup.com/info/toolkit/learn-ebm/what-is-grade/

[5] Prevention of infections which originate from vascular catheters part 2 – peripheral venous indwelling cannulas and arterial catheters recommendation by the Commission for Hospital Hygiene and Infection Prevention (KRINKO) at the Robert Koch Institute. Bundesgesundheitsblatt Gesundheitsforschung Gesundheitsschutz. 2017;60(2):207-215. doi:10.1007/s00103-016-2488-328091693

[6] Development Group of the Clinical Practice Guidelines on Intravenous Therapy with Temporary Devices in Adults. Clinical Practice Guideline on Intravenous Therapy with Temporary Devices in Adults. Madrid, Spain: Ministry of Health, Social Services and Equality; 2014.

[7] Loveday HP Wilson JA Pratt RJ. Epic3: national evidence-based guidelines for preventing healthcare-associated infections in NHS hospitals in England. J Hosp Infect. 2014;86(Suppl 1):S1-S70. doi:10.1016/S0195-6701(13)60012-2 24330862 10.1016/S0195-6701(13)60012-2 PMC7114876

[8] Royal College of Nursing. Standards for Infusion Therapy. 4th ed. London, UK: Royal College of Nursing; 2016.

[9] Nickel B Gorski L Kleidon T. Infusion therapy standards of practice. 9th ed. J Infusion Nurs. 2024;47(S1 Suppl 1):S1-S285. doi:10.1097/NAN.0000000000000532 10.1097/NAN.0000000000000532 38211609

[10] Alonso-Coella P Schünemann HJ Moberg J. GRADE Evidence to Decision (EtD) frameworks: a systematic and transparent approach to making well informed healthcare choices. 1: introduction. BMJ. 2016;353:i2016. doi:10.1136/bmj.i2016 27353417 10.1136/bmj.i2016

[11] Alonso-Coella P Oxman AD Moberg J. GRADE Evidence to Decision (EtD) frameworks: a systematic and transparent approach to making well informed healthcare choices. 2: clinical practice guidelines. BMJ. 2016;353:i2089. doi:10.1136/bmj.i2089 27365494 10.1136/bmj.i2089

[12] European Resuscitation Council. The ERC guidelines 2025 on resuscitation for everyone. Brussels: ERC; 2025. Accessed October 30, 2025. https://www.erc.edu/media/p5ymaeej/gl2025_layperson_book_ipdf-v1-e.pdf

[13] Rowley S Clare S Macqueen S. ANTT v2: an updated practice framework for aseptic technique. Br J Nurs. 2010;19(Sup1):S5-S11. doi:10.12968/bjon.2010.19.Sup1.4707920622767

[14] Thompson J Steinheiser MM Hotchkiss JB. Standards of care for peripheral intravenous catheters: evidence-based expert consensus. *J Am Assoc Vasc Access*. 2024;29(3):15-26. doi:10.2309/JAVA-D-24-00011 10.12968/bjon.2024.0422 39585227

[15] Van Rens M van der Lee R Spencer TR. The NAVIGATE project: a GloVANet-WoCoVA position statement on the nomenclature for vascular access devices. J Vasc Access. 2025;26(5):1439-1446. doi:10.1177/11297298241291248 39446461 10.1177/11297298241291248

